# Association between visceral obesity, metformin use, and recurrence risk in early-stage colorectal cancer

**DOI:** 10.1038/s41598-023-34690-y

**Published:** 2023-05-24

**Authors:** Yeshwanth Reddy Vedire, Sarbajit Mukherjee, Sumedha Dondapati, Sai Yendamuri

**Affiliations:** 1 Department of Thoracic Surgery, Roswell Park Comprehensive Cancer Center, Elm and Carlton Streets, Buffalo, NY 14263 USA; 2 Department of Medical Oncology, Roswell Park Comprehensive Cancer Center, Elm and Carlton Streets, Buffalo, NY USA

**Keywords:** Cancer, Gastrointestinal cancer

## Abstract

We sought to investigate the association between visceral obesity with disease recurrence and survival in early-stage colorectal cancer (CRC) patients. We also wanted to examine if such an association, if exists, is influenced by metformin use. Stage I/II CRC adenocarcinoma patients treated surgically were identified. L3 level CT VFI (visceral fat index) was used as a metric of visceral obesity and was calculated as the proportion of total fat area composed of visceral fat. N = 492. 53% were males, 90% were Caucasians, 35% had stage I disease, and 14% used metformin. 20.3% patients developed a recurrence over a median follow-up of 56 months. VFI was associated with both RFS and OS in a multivariate model, but not BMI. The final multivariate model for RFS included an interaction term for VFI and metformin (*p* = 0.04). Confirming this result, subgroup analysis showed an increasing VFI was associated with a poor RFS (*p* = 0.002), and OS (*p* < 0.001) in metformin non-users only and metformin use was associated with a better RFS only in the top VFI tertile (*p* = 0.01). Visceral obesity, but not BMI, is associated with recurrence risk and poorer survival in stage I/II CRC. Interestingly, this association is influenced by metformin use.

## Introduction

Colorectal cancer (CRC) is the third most common cancer in both men and women in the US^1^. Treatment of stage I and II colon cancer include surgery followed by chemotherapy in high-risk stage II patients. The current American Joint Committee on Cancer/Union Internationale Contre le Cancer (AJCC/UICC) staging system and the definition of high-risk stage II disease rely entirely on tumor characteristics. However, cancer progression undergoes a complex interaction between the host and the tumor^2,3^. As a result, we tend to either under-estimate or over-estimate the recurrence risk in early-stage colon cancer. One possible solution to this problem is to use additional biomarkers that consider patient factors.

Obesity is a major public health concern in the United States, affecting 35% of adults and 17% of children and adolescents^4^. It is an important risk factor for the development of several cancers, including colorectal cancers^5^. A recent study examined the role of diet and lifestyle-based factors, including body-mass index (BMI), on the recurrence risk in stage III colon cancer patients^6^. It found that diet and lifestyle factors can predict cancer recurrence and survival in this patient population. BMI is often used as a surrogate marker for obesity; however, this is inaccurate as it cannot differentiate between fat and lean body mass or quantify body fat ditribution^7^. Work from our group has shown that a CT imaging-based measure of obesity, the visceral fat index [VFI], is a better measure of central obesity than BMI and is associated with recurrence-free survival (RFS) and overall survival (OS) in resected stage I and stage II non-small cell lung cancer (NSCLC)^7^. In the same study, we also showed that a higher VFI was associated with tumor immuno-suppression. In another study, our group showed that the adverse effects of obesity could possibly be mitigated with the use of metformin^8^. In a cohort of four hundred thirty-four stage I NSCLC patients, Metformin use was associated with improved overall survival (OS) and disease-specific survival (DSS). However, this effect was restricted to obese patients only. This maybe due to reversal of obesity associated immune dysfunction with metformin use.

In this study, we sought to assess if this relationship is true in colon cancer as well. Therefore, we studied the association between VFI and recurrence/survival in early-stage colon cancer. We also examined if the use of metformin influences such an association.

## Results

### Cohort characteristics

After applying the inclusion and exclusion criteria, CT scans for 492 patients were identified and analyzed for visceral obesity quantification. The cohort's median age at the time of tumor diagnosis was 64 years (Inter Quartile Range [IQR] = 54–74), with a majority of the patients being male (53%). Most of the patients were white (90%). A majority of the patients consumed alcohol (62%) at the time of primary tumor diagnosis, whereas 142 (30%) and 32 (6%) patients had never consumed or consumed alcohol only in the past, respectively. There were 88 (18%), 164 (35%), and 223 (35%) current, former, and never smokers, respectively. Colon was the primary tumor site in 361 (73%) and rectum in 131 (27%) patients. 173 (35%) of the patients had stage I disease. 67 (14%) patients were metformin users. The median follow-up of the cohort was 56 months (IQR = 35–92), during which recurrence developed in 100 (20%) patients and 120 (24%) patients died. The liver was the most common site of the first recurrence, with 42 (8.5%) patients developing recurrence in the liver, followed by locoregional (6.5%), lung (3.9%), and other distant sites (1.4%). The median BMI of the cohort was 28.3 kg/m^2^ (24.3–32.7), and the median VFI and SMI were 0.44 (0.34–0.55) and 47.5 cm^2^/m^2^ (40.1–56.0), respectively. To better understand the relationship between VFI and recurrence, we categorized VFI into ‘Top’, ‘Middle’, and ‘Bottom’ tertiles based om if patients fall in the top, middle or bottom 33.33 percentile of the VFI range within the cohort.

### Univariate analysis across VFI teritiles

Univariate analysis to compare the sociodemographic and histopathological variables of CRC patients among the different VFI tertile groups revealed that patients in the top tertile were more likely to be older (top vs. middle vs. bottom, median [IQR] = 67 [58–75] vs. 66 [55–77] vs. 58 [49–67]; Kruskal Wallis rank sum test *p* value < 0.001), males (89% vs. 52% vs. 16%; Pearson’s chi-square test *p* value < 0.001), former smokers (41% vs. 39% vs. 20%; *p* < 0.001), and metformin users (18% vs. 19% vs. 4%; *p* < 0.001). Patients belonging to the top tertile were also more likely to develop recurrence (26% vs. 19% vs. 15%; *p* = 0.04). Although, a similar trend was seen with patient death (29% vs. 25% vs. 18%; *p* = 0.06), this trend was not statistically significant. There was no significant difference between patient race, alcohol consumption status, and primary tumor site and stage across VFI tertiles. BMI (29.6 [25.5–33.9] vs. 27.9 [24.4–32.1] vs. 27.2 [22.9–32.2]; *p* = 0.01) and SMI (53.5 [47.5–59.1] vs. 46.3 [39.8–55.2] vs. 42.8 [37.3–49.8]; *p* < 0.001) were significantly higher for patients in the top tertile (Table 1). Multiple linear regression modelling showed that age, gender, BMI, and alcohol history were significantly associated with VFI with β coefficients of 0.003 (Wald *p* < 0.001), − 0.17 (males vs. females; *p* < 0.001), 0.002 (*p* = 0.007), and 0.04 (never vs. former; *p* = 0.02) and 0.004 (never vs. current; *p* = 0.6) respectively, but not race or smoking history. Similar regression modelling showed that age, gender, race, and BMI were independently associated with SMI with β coefficients of − 0.18 (*p* < 0.001), − 10.70 (males vs. females; *p* < 0.001), 2.31 (white vs. non-white; *p* = 0.04), and 0.82 (*p* < 0.001) respectively, but not alcohol or smoking history.

**Table 1:** Univariate comparison of sociodemographic and histopathological variables across VFI tertiles.

Characteristic	All^a^ (n = 492)	Bottom (n = 163)	Middle (n = 161)	Top (n = 168)	*p* value^b^
Age	64 (54 -74)	58 (49–67)	66 (55–77)	67 (58–75)	**< 0. 001**
Gender					**< 0.001**
Male	260 (53%)	26 (16%)	84 (52%)	150 (89%)	
Female	232 (47%)	137 (84%)	77 (48%)	18 (11%)	
BMI	28.3 (24.3–32.7)	27.2 (22.9–32.1)	27.9 (24.4–32.1)	29.6 (25.5–33.9)	**0.01**
SMI	47.5 (40.1–56.0)	42.8 (37.3–49.8)	46.3 (39.8–55.2)	53.5 (47.5–59.1)	**< 0.001**
Race					0.4
White	444 (90%)	147 (90%)	142 (88%)	155 (92%)	
Non-White	48 (10%)	16 (10%)	19 (12%)	13 (8%)	
Smoking history					**0.001**
Current	88 (18%)	33 (20%)	25 (15%)	30 (18%)	
Former	164 (33%)	33 (20%)	62 (39%)	69 (41%)	
Never	224 (45%)	88 (54%)	68 (42%)	68 (40.5%)	
Unknown	16 (4%)	9 (6%)	6 (4%)	1 (0.5%)	
Alcohol history					0.2
Current	307 (62%)	100 (61%)	97 (60%)	110 (66%)	
Former	32 (6%)	6 (4%)	11 (7%)	15 (9%)	
Never	142 (30%)	53 (33%)	48 (30%)	41 (24%)	
Unknown	11 (2%)	4 (2%)	5 (3%)	2 (1%)	
Primary tumor site					0.6
Colon	361 (73%)	122 (75%)	114 (71%)	125 (74%)	
Rectum	131 (27%)	41 (25%)	47 (29%)	43 (26%)	
Pathological tumor stage					0.8
Stage I	173 (35%)	57 (35%)	54 (34%)	62 (37%)	
Stage II	319 (65%)	106 (65%)	107 (66%)	106 (63%)	
Metformin use					**< 0.001**
Yes	67 (14%)	7 (4%)	30 (19%)	30 (18%)	
No	425 (86%)	156 (96%)	131 (81%)	138 (82%)	
Recurrence					**0.04**
Yes	100 (20%)	25 (15%)	31 (19%)	44 (26%)	
No	392 (80%)	138 (85%)	130 (81%)	124 (74%)	
Death					0.06
Yes	120 (24%)	30 (18%)	41 (25%)	49 (29%)	
No	372 (76%)	133 (82%)	120 (75%)	119 (71%)	

Significant values are in [bold].

*BMI* body mass index, *IQR* interquartile range, *SMI* skeletal muscle index, *VFI* visceral fat index.

^a^Number and percentages, and median and interquartile range (IQR) are shown for continuous and categorical variables, respectively.

^b^Chi-square test and Kruskal Wallis rank sum test were used to compare categorical and continuous variables, respectively across VFI tertiles.

### Univariate and multivariable survival analysis

**Figure 1:**
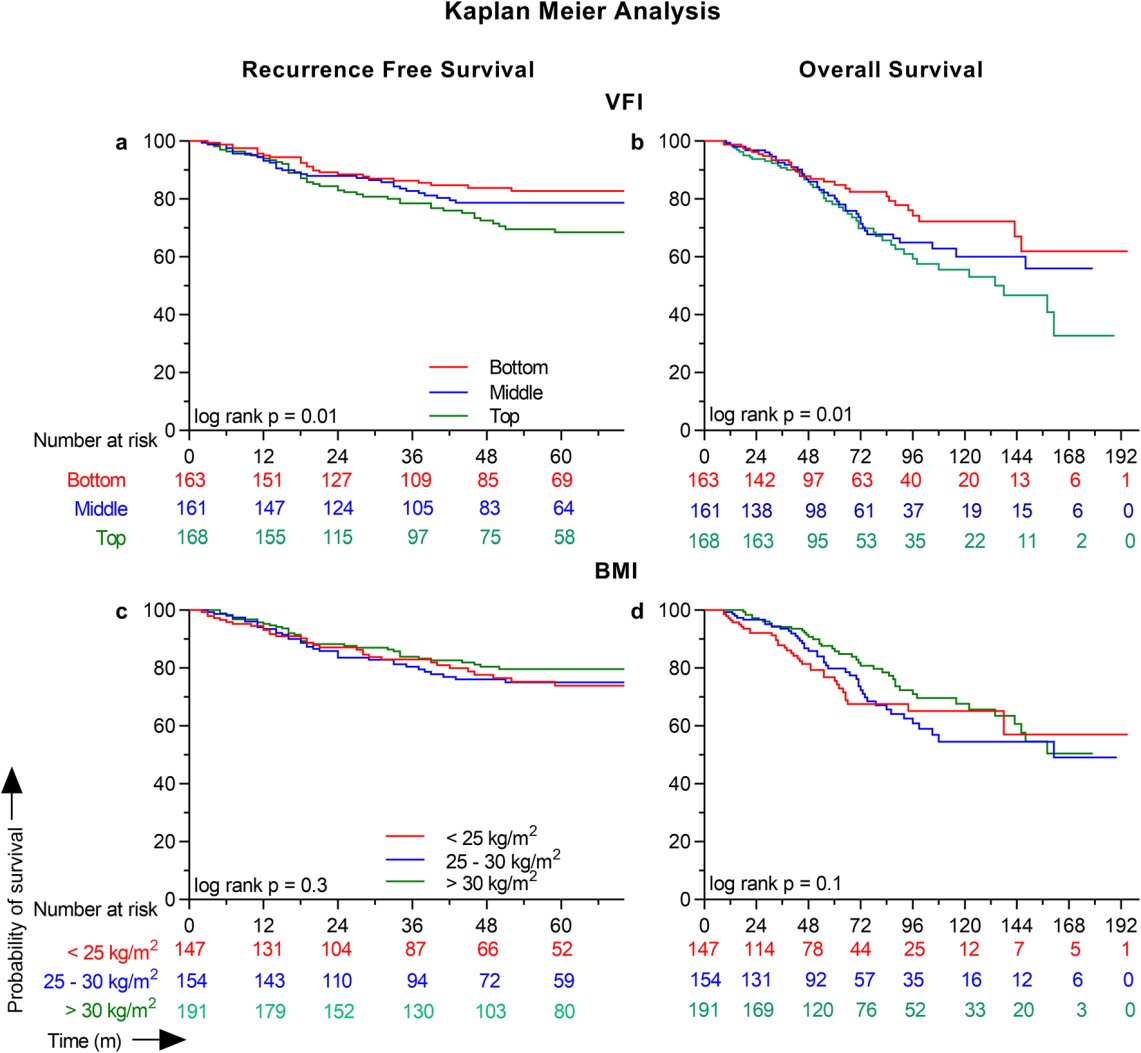
Kaplan Meier analysis of recurrence free and overall survival curves for VFI tertiles and BMI categories. Recurrence free and overall survival curves were generated for 492 patients categorized based on VFI into bottom (red), middle (blue), and top (green) teritles and BMI into < 25 kg/m^2^ (red), 25–30 kg/m^2^ (blue), and > 30 kg/m^2^ (green) categories. Kaplan Meier survival curve analysis revealed that higher VFI had poorer RFS (log rank *p* = 0.01) and OS (*p* = 0.01), but not BMI.

**Figure 2:**
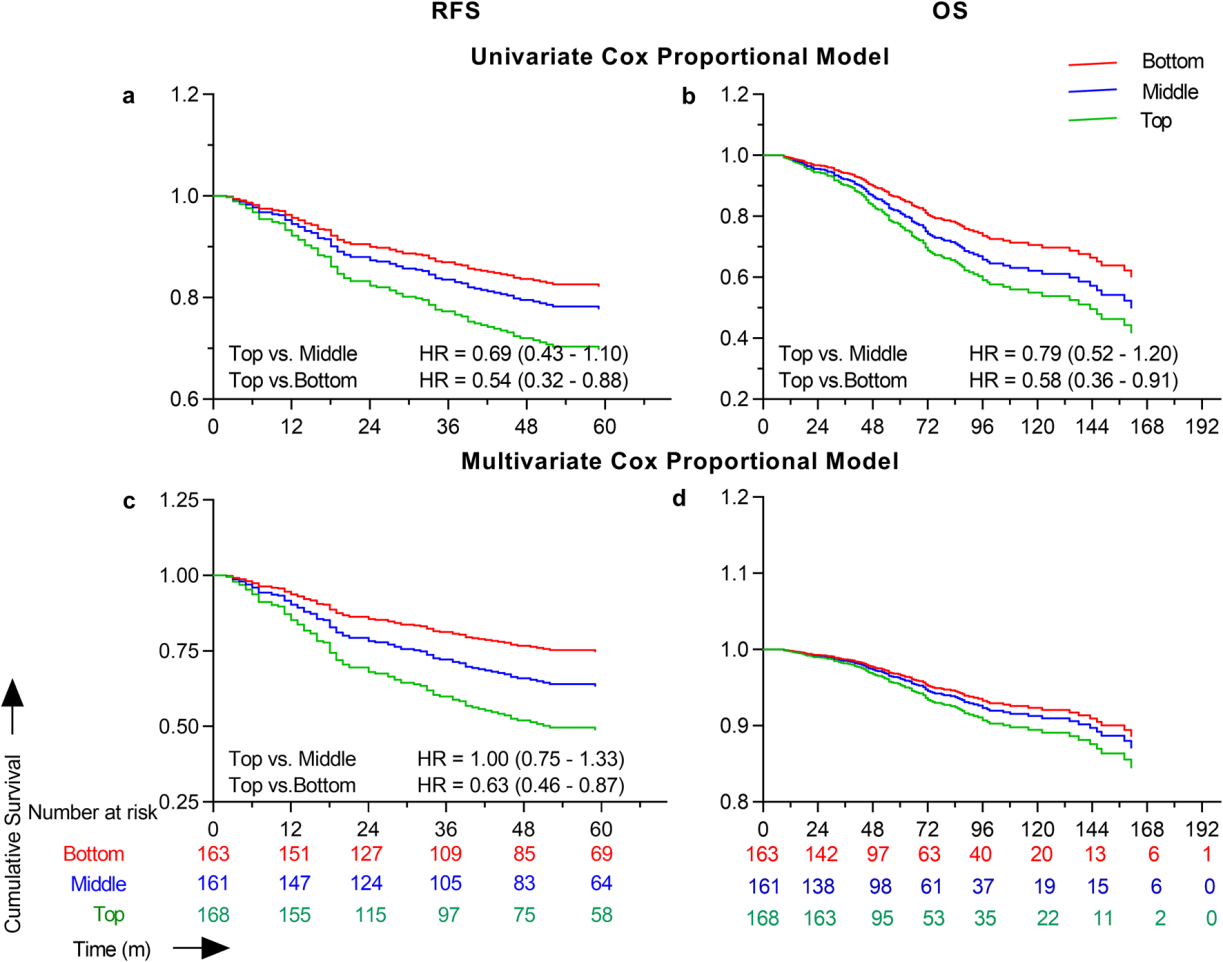
Univariate and multivariate cox proportional models of VFI tertiles for overall and recurrence free survival. Univariate and multivariate cox proportional model survival curves were generated for 492 patients based on the bottom (red), middle (blue), and top (green) VFI tertiles. Multivariate analysis was performed with patient age, sex, tumor stage, and VFI as covariates for RFS. Patient age, sex, race, and VFI were used as covariates in the multivariate model for OS. Univariate (Wald *p* = 0.01) and multivariate (*p* = 0.005) analysis showed that a bottom VFI tertile was associated with a significantly better recurrence free survival. Univariate analysis showed that the bottom VFI tertile (*p* = 0.01) was associated with a significantly better OS. However, VFI lost its significance and was not included in the final multivariate model for OS.

Time to event survival analysis performed using univariate Cox proportional modelling showed that increasing patient age (HR [95% C] = 0.98 [0.96–0.99]; Wald *p* = 0.01), female sex (0.55 [0.36–0.82]; *p* = 0.004), and stage I tumor (0.54 [0.33–0.85]; *p* = 0.009) were significant prognostic indicators for risk of recurrence within 5 years. Patient race, smoking and alcohol history, primary tumor site, metformin use, and BMI were not associated with RFS on univariate analysis. However, every unit increase in VFI showed a significant increase in the risk of recurrence with a HR of 5.04 (1.24–20.57; *p* = 0.02). Similarly, the bottom (0.54 [0.32–0.88]; *p* = 0.02) and middle (0.69 [0.43–1.10]; *p* = 0.1) VFI tertiles were associated with a decreased risk of recurrence compared to the top tertile (Fig. 1), but BMI categories were not. Similar to VFI, every 1 cm^2^/m^2^ increase in SMI was associated with a HR of 1.02 (1.00–1.03; *p* = 0.01). Multivariate Cox proportional modeling for RFS was performed with patient age, tumor stage as their respective univariate *p* was < 0.2, and either BMI or SMI, or VFI as covariates in different models using the stepwise backward LR model. Multivariate analysis retained only age (0.97 [0.95–0.98]; *p* = 0.001), tumor stage I (0.54 [0.34–0.86], *p* = 0.009), and VFI (12.75 [2.88–56.45], *p* = 0.001) as the significant prognostic indicators in the final model for RFS. Confirming this result, when VFI was analyzed as a categorical variable, the bottom and middle tertiles had HRs of 0.63 (0.46–0.87; *p* = 0.005) and 1.00 (0.75–1.33; *p* = 0.9) compared to the top tertile (Fig. 2). Neither BMI nor SMI was found to be prognostic for RFS as they were not included in the final model of the multivariate analysis (Table 2). Similar survival analysis performed after excluding 186 patients who received adjuvant chemo- or radiotherapy (n = 306) to adjust for confounding showed that patients in the top VFI tertile had significantly poorer RFS compared to patients in the bottom tertile (Supplementary Table S1).

**Table 2:** Results of univariate and multivariate cox proportional models for recurrence free and overall survival.

Variable	Recurrence free survival		Overall survival	
	Univariate HR (95% CI)	Multivariate HR (95% CI)^a^	Univariate HR (95% CI)	Multivariate HR (95% CI)^b^
Age	**0.98**	**0.97**	**1.02**	**1.02**
	**(0.96–0.99)**	**(0.95–0.98)**	**(1.01–1.04)**	**(1.00–1.03)**
Gender				
Female versus male	**0.55**		0.73	
	**(0.36–0.82)**		(0.50–1.04)	
BMI	0.99		0.97	0.97
	(0.96–1.02)		(0.94–1.00)	(0.94–1.00)
SMI	**1.02**		0.99	
	**(1.00–1.03)**		(0.98–1.01)	
Race				
Non-white versus White	1.13		**1.74**	**1.39**
	(0.57–2.03)		**(1.03–2.77)**	**(1.09–1.78)**
Smoking history				
Current versus never	1.16		1.14	
	(0.68–1.92)		(0.69–1.84)	
Former versus never	1.01		1.21	
	(0.64–1.57)		(0.80–1.84)	
Alcohol history				
Current versus never	1.19		1.05	
	(0.77–1.89)		(0.70–1.59)	
Former versus never	1.07		1.21	
	(0.40–2.42)		(0.56–2.35)	
Primary tumor site				
Colon versus rectum	0.7		0.8	
	(0.47–1.07)		(0.55–1.18)	
Pathological tumor stage				
Stage I versus stage II	**0.54**	**0.54**	0.79	
	**(0.33–0.85)**	**(0.34–0.86)**	(0.53–1.15)	
Metformin				
Use versus non-use	0.7		0.79	
	(0.35–1.26)		(0.46–1.34)	
VFI	**5.04**	**12.75**	**8.55**	**4.95**
	**(1.24–20.57)**	**(2.88–56.45)**	**(2.30–32.35)**	**(1.21–20.16)**
VFI tertiles				
Middle versus top	0.69	1	0.79	
	(0.43–1.10)	(0.75–1.33)	(0.52–1.20)	
Bottom versus top	**0.54**	**0.63**	**0.58**	
	**(0.32–0.88)**	**(0.46–0.87)**	**(0.36–0.91)**	

*BMI* body mass index, *CI* confidence interval, *HR* hazard ratio, *IQR* interquartile range, *SMI* skeletal muscle index, *VFI* visceral fat index.

^a^Multivariate analysis for RFS was performed using covariates with a univariable *p* value < 0.2, which included patient age, sex, tumor stage, and VFI/SMI/BMI. Only variables that were included in the final multivariable model are shown. VFI as a categorical variable, BMI, and SMI were analyzed in different models but shown in the same table for ease. All statistically significant variables (*p* < 0.05) are in bold.

^b^Multivariate analysis for OS was performed using covariates with univariable *p* value < 0.2, which included patient age, sex, race, and VFI/SMI/BMI. Only variables that were included in the final multivariable model are shown. VFI as a categorical variable, BMI, and SMI were analyzed in different models but shown in the same table for ease. All statistically significant variables (*p* < 0.05) are in bold.

A similar time to event analysis with overall survival (OS) showed that non-white patient race (1.74 [1.03–2.77]; *p* = 0.02), increasing age (1.02 [1.01–1.04]; *p* = 0.0004) and VFI (8.55 [2.30–32.35]; *p* = 0.001) were associated with poor OS on univariate Cox proportional modelling. As was seen with RFS, smoking and alcohol history, primary tumor site, metformin use, and BMI were not significantly associated with OS. In addition to the above variables, tumor stage and SMI were not associated with OS. Similar to univariate analysis, multivariate analysis of OS retained age (1.02 [1.00–1.03]; *p* = 0.004), non-white race (1.39 [1.09–1.78]; *p* = 0.004), and VFI (4.95 [1.21–20.16]; *p* = 0.02) in the final model. VFI as tertiles was not associated with OS in either univariate or multivariate analyses (Table 2; Fig. 2).

In the above analysis we analyzed patients with colon (N = 361 [73%]), and rectum (N = 161 [275]) as the primary tumor sites. To potentially account for the differing treatment approaches in patients based on the primary tumor site, we repeated the above analyses as subgroup analysis based on primary tumor site^9,10^. We found that similar to the entire cohort, age, pathological stage, and VFI were retained in the final multivariate model for RFS, whereas, age, race, and VFI were retained for OS as prognostic indicators in patients with colon as the primary site (Supplementary Table S2). A similar analysis in rectal cancer patients did not show any significant results potentially due to the low power in the analysis given the small sample size. Additionally, we performed another analysis including the number of lymph nodes examined, if patients received adjuvant/neoadjuvant chemotherapy or not, and using pathological T stage instead of pathological stage I or II. This additional analysis confirmed our observations with VFI as a significant prognostic indicator for both RFS, and OS (Supplementary Table S3).

### Metformin and VFI interaction and subgroup analysis

Our previous work showed a significant interaction between metformin and obesity when assessing outcomes in lung cancer patients^8^. We sought to assess if a similar interaction between VFI and metformin existed in colorectal cancer by introducing an interaction variable between VFI and metformin use to the multivariate model. This modeling showed that this was indeed the case, whether VFI was used as a continuous or categorical (tertiles) variable. Variables retained in the final model are highlighted in Table 3. A similar analysis for OS did not retain the metformin/VFI interaction variable in the final model. To further explore the interaction between metformin use and visceral obesity, a subgroup analysis examining the association between metformin use and RFS in each of the VFI tertiles was performed. Similarly, the relationship between VFI and outcome in metformin users and non-users was also assessed (Supplementary Table S1). Interestingly, increasing VFI was associated with a significantly poor RFS (10.20 [2.28–45.55]; *p* = 0.002), and OS (16.74 [3.98–70.44]; *p* < 0.001) in metformin non-users, but not metformin users (Supplementary Fig. S1). Metformin use was associated with a significantly better RFS only in the top VFI tertile (0.24 [0.07–0.79]; *p* = 0.01). However, a similar OS benefit was not observed with metformin use in any other VFI tertile (Supplementary Fig. S2).

**Table 3:** Results of multivariate Cox proportional models for recurrence free and overall survival with a VFI and metformin interaction variable.

Variable	Recurrence free survival		Overall survival	
	Multivariate HR (95%CI)^a^	*p* value	Multivariate HR (95%CI)^b^	*p* value^c^
Age	**0.97**	**0.001**	**1.02**	**0.003**
	**(0.95–0.98)**		**(1.00–1.03)**	
Race				
Non-White versus White			**1.43**	**0.005**
			**(1.11–1.85)**	
Pathological tumor stage				
Stage I versus stage II	**0.54**	**0.009**		
	**(0.34–0.86)**			
Metformin				
Use versus non-use	11.77	0.07	3.99	0.2
	(0.77–177.72)		(0.30–52.51)	
VFI	**26.61**	**< 0.001**	**10.39**	**0.002**
	**(5.38–131.48)**		**(2.30–46.97)**	
VFI: metformin		**0.04**		0.1
VFI tertiles				
Middle versus top	**0.51**	**0.01**		
	**(0.31–0.85)**			
Bottom versus top	**0.34**	**< 0.001**		
	**(0.20–0.59)**			
VFI tertile: metformin		**0.03**		

*CI* confidence interval, *HR* hazard ratio, *IQR* interquartile range, *VFI* visceral fat index.

^a^Multivariate analysis for RFS was performed using covariates with a univariable *p* value < 0.2, which included age, sex, tumor stage, VFI, and metformin and an interaction variable between VFI and metformin. Only variables that were included in the final multivariate model are shown. VFI as a categorical variable and its interaction with metformin was analyzed in a different model but shown in the same table for ease. All statistically significant variables (*p* < 0.05) are in bold.

^b^Multivariate analysis for OS was performed using covariates with univariable *p* value < 0.2, which included age, sex, race, VFI, and metformin and an interaction variable between VFI and metformin. Only variables that were included in the final multivariate model are shown. VFI as a categorical variable and its interaction with metformin was analyzed in a different model but shown in the same table for ease. All statistically significant variables (*p* < 0.05) are in bold.

^c^Multivariate Cox proportional Wald *p* values. All statistically significant Wald *p* values (*p* < 0.05) are in bold.

## Discussion

A European study suggests that obesity is responsible for around 11% of colorectal cancer (CRC) cases^11^. Our study showed that higher VFI as a metric of visceral obesity is associated with worse RFS and OS. Our findings are similar to that of Fleming et al., who showed that a high visceral to total fat ratio was associated with poor clinical and oncological outcomes in non-metastatic CRC^12^. Interestingly, patients with a high visceral to total fat ratio also had higher levels of IL-6 and tumor necrosis factor α, suggesting that the detrimental effects of obesity may be mediated by inflammation. It has been widely accepted that obesity leads to a chronic inflammatory state which in turn leads to immune dysfunction and the development of cancers^13^. We recently showed that obesity enhances PD-1 mediated T-cell dysfunction at least partly by leptin signaling^14^. This could potentially explain why patients with higher VFI in our study had worse outcomes.

An important question in obesity-related research is how obesity is defined. Because of its ease of calculation, BMI has been traditionally used to define obesity. In a study, Sinicrope et al. showed that a BMI ≥ 35.0 kg/m^2^ statistically significantly reduced DFS compared with normal-weight patients in men. However, this adverse effect of obesity was not found in females^15^. However, previous reports have mentioned that visceral obesity may be a more important predictor of outcomes in CRC patients than BMI^16^. We did not see an association between BMI and either RFS or OS in our study. In contrast to Sinicrope et al. gender was not statistically significant in our multivariate model. As explained before, this could be due to BMI's inability to adjust for body composition, contributing to what is known as the “obesity paradox”. Of note, unlike the findings by Fleming et al. we did not see any association between skeletal muscle content and recurrence or survival^12^. These could be due to differences in the study population, measurement of SMI, stage distribution, or other confounding factors.

Another interesting finding of this study was that the association between body composition and CRC recurrence is affected by metformin use. Consistent with our previous research in NSCLC, we showed that the detrimental effect of high VFI is only present in non-metformin users. Similarly, the protective effect of metformin was only present in the high VFI group^7^. The possible mechanisms may be AMPK (5′-AMP-activated protein kinase) mediated cell cycle arrest^17^, inhibition of reactive oxygen species generation by inhibiting the Electron Transport Chain^18^, promotion of apoptosis and autophagy of tumor cells^19^, and inhibition of leptin induced T cell exhaustion^20^ and increased number of CD3+ and CD8+ tumor infiltrating lymphocytes^21^ by metformin. The possible interaction between obesity, metformin, and survival in colorectal cancer could be the reversal of the metabolic and immune dysfunction by metformin within the tumor microenvironment in the high VFI group through the above mechanisms.

However, this interaction between visceral obesity, metformin use, and recurrence was not observed in overall survival. This discrepancy between RFS and OS was also observed by Sauer et al. when comparing preoperative and postoperative chemotherapy in rectal cancer patients^22^. Similarly, Andre et al. found that adjuvant chemotherapy improved only disease free survival and not OS^23^. Possible explanations for this discrepancy could be that the follow up period is small to detect a significant difference, and patient comorbidities and other cofounding variables which might affect OS but not RFS.

To our knowledge, this is the first study that established an association between visceral obesity, metformin use, and clinical and oncologic outcomes in early-stage CRC. However, our study has several limitations, including the retrospective design, relatively small sample size limiting the power of the study, lack of detailed information on treatment, patient co-morbidities, and molecular characteristics of tumors. Also, the dose and duration of metformin were not available. Nevertheless, our findings are hypothesis-generating and consistent with our previous findings that metformin can reverse obesity-induced immune exhaustion. Prospective clinical studies and animal experiments are needed to validate our hypothesis.

## Methods

### Institutional review board and informed consent statement

This study was conducted in accordance with the Declaration of Helsinki and approved by the Institutional Review Board of Roswell Park Comprehensive Cancer Center. Informed consent requirement was waived by the institutional review board.

### Clinical data

All patients with Stage I/II colorectal adenocarcinoma undergoing surgery at our institute between 2004 and 2020 were included. Institution databases, cancer registries, and electronic health records were used to extract clinical data. Information on patient age, gender, race (Caucasian, African American, other), smoking status (current, former, and never), alcohol consumption (current, former, and never), height, BMI, metformin use, primary tumor site (caecum, colon, rectosigmoid, and rectum) and pathological T and N stage (as per the 7th or 8th editions of the staging manual of the American Joint Committee on Cancer) at the time of diagnosis, number of lymph nodes examined, received adjuvant therapy or not, tumor recurrence, site (locoregional, liver, lung, and other distant sites), and recurrence-free and overall survival data was extracted. Patients diagnosed with a primary tumor in the appendix and who developed a recurrence > 5 years after primary tumor diagnosis were excluded. Data was collapsed into white (Caucasians) and non-white (African American and others) for patient race, colon (caecum and colon), and rectum (rectosigmoid and rectum) as primary tumor sites for analysis.

### CT imaging analysis

This work was performed by authors YRV and SD (Interobserver reliability statistics are explained in Supplementary Text S1). CT scans that were obtained for staging studies, preoperative workup, or surveillance postoperatively were obtained. A single axial cross-sectional image at the L3 vertebral level was identified for analysis. Patients whose entire body cross-sectional axial images within 5 years of primary tumor diagnosis could not be identified were excluded. To quantify visceral adiposity and muscle mass, sliceOmatic software (version 5.0 Rev-16c; Tomovision Software, Magog, Canada) with ABACS+ plugin (Rev-1.0.0; Voronoi Health Analytics, Vancouver, Canada) was used. The images are labeled according to their vertebral level (L3) prior to auto-segmentation of image pixels using the *Vertebral Label* tool. The ABACS+ plugin performs segmentation using preset Hounsfield Unit (HU) ranges for muscle, bone, visceral, subcutaneous, and intramuscular adipose tissues. After segmentation by ABACS+, the *Region Growing* mode and *Tag Lock* tool were used to make necessary manual corrections using the preset HU values in the *Alberta L3 Manual Protocol* available as an additional download. The *Tag Surface/Volume* tool was used to calculate and export the surface area measurements of muscle, bone, visceral, subcutaneous, and intramuscular adipose tissue. The actual cross-sectional visceral fat area (VFA) was calculated as the sum of visceral and intramuscular adipose tissue areas (True visceral = Visceral + Intramuscular). The total cross-sectional fat area (TFA) was calculated as the sum of true visceral and subcutaneous adipose tissue areas (Total fat area = True visceral + Subcutaneous). Visceral obesity was quantified as an index (visceral fat index, VFI), calculated as the ratio of VFA to TFA (VFA/TFA). The SMI (skeletal muscle index) was calculated after adjusting the L3 cross-sectional skeletal muscle area to the square of the patient’s height (muscle area [cm^2^]/height^2^ [m]) as a measure of the total body muscle mass.

### Statistical analysis

Since VFI has no established cut-off values, analysis was performed using it as both a continuous and categorical variable as tertiles. Kruskal Wallis rank-sum test and Pearson’s chi-square test were used for group comparisons of continuous and categorical variables, respectively. Survival analysis was performed using Kaplan Meier survival curve analysis and Cox proportional modeling. For multivariate Cox proportional modeling, covariates with a *p* value < 0.2 on univariate cox modeling were used in addition to BMI, SMI, or VFI as covariates. A Backward LR stepwise method with *p* < 0.1 as tolerance at each step was used to develop a final model. To understand if an interaction between metformin and obesity exists, as shown by us in lung cancer previously, an interaction analysis followed by a subgroup analysis was performed based on VFI tertiles and metformin use^8^. An alpha error of 0.05 was used to assess statistical significance. SPSS (version 26, IBM Software, Armonk, NY) and Prism (version 9.3.1 for Windows OS, GraphPad Software, San Diego, CA) were used for graphing and analysis.

### Other

This study is reported as per Strengthening the Reporting of Observational Studies in Epidemiology (STROBE) guidelines for cohort studies (Supplementary Table S4).

### Ethical statement

This retrospective study was approved by the institutional review board of Roswell Park Comprehensive Cancer Center (RPCCC), Buffalo, New York, USA.

## Supplementary Information

Supplementary Information.

## Data Availability

Raw data generated in this study is available from the authors upon request.
