# Supplementary material for: Association between visceral obesity, metformin use, and recurrence risk in early-stage colorectal cancer
Source: Sci Rep. 2023 May 24;13:8401. doi: 10.1038/s41598-023-34690-y (PMC10209144; doi:10.1038/s41598-023-34690-y)
Supplement: Supplementary file 1 — Supplementary Information. [file 41598_2023_34690_MOESM1_ESM.docx]

**Supplemental Material**

Association between visceral obesity, metformin use, and recurrence risk in early-stage colorectal cancer.

*Content*

1. Supplementary Table S1. Results of univariate and multivariate cox proportional models for recurrence free and overall survival after excluding patients who received adjuvant therapy (n = 306).
2. Supplementary Table S2. Results of univariate and multivariate cox proportional models for recurrence free and overall survival in patients with colon as primary tumor site (n = 361).
3. Supplementary Table S3. Results of univariate and multivariate cox proportional models for recurrence free and overall survival in patients including additional variables (n = 492).
4. Supplementary Figure S1. Univariate Cox proportional models of VFI tertiles for recurrence free and overall survival in metformin users and non-users
5. Supplementary Figure S2. Univariate Cox proportional models based on metformin use for recurrence free and overall survival in patients belonging to different VFI tertiles
6. Supplementary Table S4. STROBE statement–Checklist of items that should be included in reports of cohort studies

**Supplementary Table S1**. Results of univariate and multivariate cox proportional models for recurrence free and overall survival after excluding patients who received adjuvant therapy (n = 306).

| **Variable** | **Recurrence Free Survival**  **(n = 306)** | | **Overall Survival**  **(n = 306)** | |
| --- | --- | --- | --- | --- |
|  | **Univariate HR (95% CI)** | **Multivariate HR (95% CI)^a^** | **Univariate HR (95% CI)** | **Multivariate HR (95% CI)^b^** |
| **Age** | 0.97 | 0.98 | **1.02** | **1.01** |
|  | (0.95 - 1.00) | (0.96 - 1.00) | **(1.00 - 1.04)** | **(1.00 - 1.03)** |
| **Gender** |  |  |  |  |
| Female vs. Male | **0.50** | **0.51** | 0.77 |  |
|  | **(0.26 - 0.92)** | **(0.27 – 0.94)** | (0.47 - 1.26) |  |
| **BMI** | 0.99 |  | **0.94** |  |
|  | (0.94 - 1.04) |  | **(0.90 - 0.99)** |  |
| **SMI** | 1.01 |  | 0.97 |  |
|  | (0.98 - 1.04) |  | (0.95 - 1.00) |  |
| **Race** |  |  |  |  |
| Non-white vs. White | 1.09 |  | 1.55 |  |
|  | (0.37 – 2.52) |  | (0.71 - 3.00) |  |
| **Smoking History** |  |  |  |  |
| Current vs. Never | 1.23 |  | 1.69 |  |
|  | (0.53 - 2.62) |  | (0.85 - 3.23) |  |
| Former vs. Never | 0.94 |  | 1.40 |  |
|  | (0.47 - 1.85) |  | (0.78 - 2.49) |  |
| **Alcohol History** |  |  |  |  |
| Current vs. Never | 1.17 |  | 0.88 |  |
|  | 0.61 – 2.38) |  | (0.51 - 1.56) |  |
| Former vs. Never | 1.41 |  | 1.75 |  |
|  | (0.32 – 4.45) |  | (0.68 - 3.97) |  |
| **Primary Tumor Site** |  |  |  |  |
| Colon vs. Rectum | 1.18 |  | 1.13 |  |
|  | (0.54 - 3.12) |  | (0.61 - 2.31) |  |
| **Pathological Tumor Stage** |  |  |  |  |
| Stage I vs. Stage II | **0.43** | **0.42** | 0.61 |  |
|  | **(0.20 - 0.83)** | **(0.21 - 0.84)** | (0.35 - 1.02) |  |
| **Metformin** |  |  |  |  |
| Use vs. Non-use | 0.77 |  | 0.91 |  |
|  | (0.29 - 1.69) |  | (0.46 - 1.65) |  |
| **VFI** | 2.74 |  | 3.81 |  |
|  | (0.34 -22.15) |  | (0.68 - 22.08) |  |
| **VFI Tertiles** |  |  |  |  |
| Middle vs. Top | 0.65 | 1.08 | 0.99 |  |
|  | (0.31 - 1.29) | (0.69 - 1.69) | (0.55 -1.75) |  |
| Bottom vs. Top | **0.47** | **0.55** | 0.71 |  |
|  | **(0.21 - 0.97)** | **(0.33 - 0.91)** | (0.37 - 1.32) |  |

^a^ Multivariable analysis for RFS (n = 306) was performed after excluding 186 patients who received adjuvant chemo- or radiotherapy using covariates with a univariable p-value < 0.2, which included patient age, sex, tumor stage, and VFI/SMI/BMI. Only variables that were included in the final multivariable model are shown. VFI as a categorical variable was analyzed in a different model but shown in the same table for ease. All statistically significant variables (p < 0.05) are in bold.

^b^ Multivariable analysis for OS (n = 306) was performed after excluding 186 patients who received adjuvant chemo- or radiotherapy using covariates with univariable p-value < 0.2, which included patient age, and stage and VFI/SMI/BMI. Only variables that were included in the final multivariable model are shown. VFI as a categorical variable was analyzed in a different model but shown in the same table for ease. All statistically significant variables (p < 0.05) are in bold.

BMI, body mass index; CI, confidence interval; HR, hazard ratio; SMI, skeletal muscle index; VFI, visceral fat index

**Supplementary Table S2**. Results of univariate and multivariate cox proportional models for recurrence free and overall survival in patients with colon as primary tumor site (n = 361).

| **Variable** | **Recurrence Free Survival** | | **Overall Survival** | |
| --- | --- | --- | --- | --- |
|  | **Univariate HR (95% CI)** | **Multivariable HR (95% CI)^a^** | **Univariate HR (95% CI)** | **Multivariable HR (95% CI)^b^** |
| **Age** | **0.98** | **0.97** | **1.02** | **1.02** |
|  | **(0.96 - 0.99)** | **(0.95 - 0.99)** | **(1.00 - 1.04)** | **(1.01 - 1.04)** |
| **Gender** |  |  |  |  |
| Female vs. Male | **0.46** |  | 0.78 |  |
|  | **(0.28 - 0.78)** |  | (0.50 - 1.23) |  |
| **BMI** | 0.99 |  | 0.97 | 0.97 |
|  | (0.95 - 1.03) |  | (0.94 - 1.00) | (0.94 - 1.00) |
| **SMI** | **1.02** |  | 0.99 |  |
|  | **(1.00 - 1.04)** |  | (0.98 - 1.01) |  |
| **Race** |  |  |  |  |
| Non-white vs. White | 1.15 |  | 1.69 |  |
|  | (0.55 - 2.42) |  | (0.93 - 3.08) |  |
| **Smoking History** | |  |  |  |
| Current vs. Never | 1.21 |  | 1.41 |  |
|  | (0.63 - 2.31) |  | (0.78 - 2.53) |  |
| Former vs. Never | 1.05 |  | 1.15 |  |
|  | (0.60 - 1.84) |  | (0.68 - 1.95) |  |
| **Alcohol History** | |  |  |  |
| Current vs. Never | 1.02 |  | 1.01 |  |
|  | (0.60 - 1.75) |  | (0.62 - 1.67) |  |
| Former vs. Never | 0.96 |  | 1.13 |  |
|  | (0.33 - 2.83) |  | (0.46 - 2.78) |  |
| **Pathological T Stage** | |  |  |  |
| Stage T1 &T2 vs. Stage T4 | **0.21** | **0.26** | **0.29** |  |
|  | **(0.10 - 0.43)** | **(0.12 - 0.54)** | **(0.15 - 0.54)** |  |
| Stage T3 vs. T4 | **0.32** | **0.41** | **0.25** |  |
|  | **(0.18 - 0.57)** | **(0.23 - 0.72)** | **(0.14 - 0.43)** |  |
| **Number of Lymph Nodes Examined** | **0.94** | **0.94** | 0.99 |  |
|  | **(0.91 - 0.96)** | **(0.92 - 0.97)** | (0.97 - 1.00) |  |
| **Adjuvant Therapy Received** | |  |  |  |
| No vs. Yes | **0.55** |  | 1.22 |  |
|  | **(0.34 - 0.91)** |  | (0.75 - 1.99) |  |
| **Metformin** |  |  |  |  |
| Use vs. Non-use | 1.5 |  | 1.36 |  |
|  | (0.64 - 3.48) |  | (0.67 - 2.73) |  |
| **VFI** | **9.26** | **19.59** | **10.77** | **4.57** |
|  | **(1.64 - 52.06)** | **(3.22 - 119.09)** | **(2.04 - 56.77)** | **(1.12 - 18.65)** |
| **VFI Tertiles** | |  |  |  |
| Middle vs. Top | 0.62 | 0.59 | 0.71 |  |
|  | (0.35 - 1.11) | (0.33 - 1.05) | (0.42 - 1.21) |  |
| Bottom vs. Top | **0.44** | **0.36** | **0.54** |  |
|  | **(0.24 - 0.82)** | **(0.18 - 0.69)** | **(0.31 - 0.96)** |  |

^a^ Multivariable analysis for RFS (n = 361) was performed after excluding 173 patients who had a rectal primary tumor site using covariates with a univariable p-value < 0.2, which included patient age, sex, pathological T stage, lymph nodes examined and VFI/SMI/BMI. Only variables that were included in the final multivariable model are shown. VFI as a categorical variable was analyzed in a different model but shown in the same table for ease. All statistically significant variables (p < 0.05) are in bold.

^a^ Multivariable analysis for OS (n = 361) was performed after excluding 173 patients who had a rectal primary tumor site using covariates with a univariable p-value < 0.2, which included patient age, race, and VFI/SMI/BMI. Only variables that were included in the final multivariable model are shown. VFI as a categorical variable was analyzed in a different model but shown in the same table for ease. All statistically significant variables (p < 0.05) are in bold.

BMI, body mass index; CI, confidence interval; HR, hazard ratio; SMI, skeletal muscle index; VFI, visceral fat index

**Supplementary Table S3**. Results of univariate and multivariate cox proportional models for recurrence free and overall survival in patients including additional variables (n = 492).

| **Variable** | **Recurrence Free Survival** | | **Overall Survival** | |
| --- | --- | --- | --- | --- |
|  | **Univariate HR (95% CI)** | **Multivariable HR (95% CI)^a^** | **Univariate HR (95% CI)** | **Multivariable HR (95% CI)^b^** |
| **Age** | **0.98** | **0.97** | **1.02** | **1.02** |
|  | **(0.96 - 0.99)** | **(0.96 - 0.99)** | **(1.01 - 1.04)** | **(1.01 - 1.04)** |
| **Gender** |  |  |  |  |
| Female vs. Male | **0.55** |  | 0.73 |  |
|  | **(0.36 - 0.82)** |  | (0.50 - 1.04) |  |
| **BMI** | 0.99 |  | 0.97 | 0.97 |
|  | (0.96 - 1.02) |  | (0.94 - 1.00) | (0.94 - 1.00) |
| **SMI** | **1.02** |  | 0.99 |  |
|  | **(1.00 - 1.03)** |  | (0.98 - 1.01) |  |
| **Race** |  |  |  |  |
| Non-white vs. White | 1.13 |  | **1.74** | **1.39** |
|  | (0.57 – 2.03) |  | **(1.03 - 2.77)** | **(1.08 - 1.79)** |
| **Smoking History** | |  |  |  |
| Current vs. Never | 1.16 |  | 1.14 |  |
|  | (0.68 - 1.92) |  | (0.69 - 1.84) |  |
| Former vs. Never | 1.01 |  | 1.21 |  |
|  | (0.64 - 1.57) |  | (0.80 - 1.84) |  |
| **Alcohol History** | |  |  |  |
| Current vs. Never | 1.19 |  | 1.05 |  |
|  | (0.77 - 1.89) |  | (0.70 - 1.59) |  |
| Former vs. Never | 1.07 |  | 1.21 |  |
|  | (0.40 - 2.42) |  | (0.56 - 2.35) |  |
| **Primary Tumor Site** | |  |  |  |
| Colon vs. Rectum | 0.7 |  | 0.8 |  |
|  | (0.47 - 1.07) |  | (0.55 - 1.18) |  |
| **Pathological T Stage** | |  |  |  |
| Stage T1 &T2 vs. Stage T4 | **0.27** | **0.53** | **0.68** |  |
|  | **(0.155 - 0.48)** | **(0.39 - 0.73)** | **(0.52 - 0.88)** |  |
| Stage T3 vs. T4 | **0.41** | 0.94 | **0.67** |  |
|  | **(0.25 - 0.67)** | (0.72 - 1.23) | **(0.53 - 0.85)** |  |
| **Number of Lymph Nodes Examined** | **0.95** | **0.94** | 0.99 |  |
|  | **(0.93 - 0.96)** | **(0.93 - 0.96)** | (0.98 - 1.00) |  |
| **Adjuvant Therapy Received** | |  |  |  |
| No vs. Yes | **0.49** |  | 0.97 |  |
|  | **(0.33 - 0.72)** |  | (0.81 - 1.16) |  |
| **Metformin** |  |  |  |  |
| Use vs. Non-use | 0.7 |  | 0.79 |  |
|  | (0.35 - 1.26) |  | (0.46 - 1.34) |  |
| **VFI** | **5.04** | **9.49** | **8.55** | **4.57** |
|  | **(1.24 -20.57)** | **(2.10 - 42.90)** | **(2.30 - 32.35)** | **(1.12 - 18.65)** |
| **VFI Tertiles** |  |  |  |  |
| Middle vs. Top | 0.69 | 0.94 | 0.79 |  |
|  | (0.43 - 1.10) | (0.71 - 1.26) | (0.52 -1.20) |  |
| Bottom vs. Top | **0.54** | **0.69** | **0.58** |  |
|  | **(0.32 - 0.88)** | **(0.50 - 0.95)** | **(0.36 - 0.91)** |  |

^a^ Multivariable analysis for RFS was performed using covariates with a univariable p-value < 0.2, which included patient age, sex, pathological T stage, lymph nodes examined and VFI/SMI/BMI. Only variables that were included in the final multivariable model are shown. VFI as a categorical variable was analyzed in a different model but shown in the same table for ease. All statistically significant variables (p < 0.05) are in bold.

^a^ Multivariable analysis for OS was performed using covariates with a univariable p-value < 0.2, which included patient age, race, and VFI/SMI/BMI. Only variables that were included in the final multivariable model are shown. VFI as a categorical variable was analyzed in a different model but shown in the same table for ease. All statistically significant variables (p < 0.05) are in bold.

BMI, body mass index; CI, confidence interval; HR, hazard ratio; SMI, skeletal muscle index; VFI, visceral fat index

**

**

**Supplementary Figure S1.** *Univariate Cox proportional models of VFI tertiles for recurrence free and overall survival in metformin users and non-users.* Univariate Cox proportional model survival curves were generated for 67 and 425 metformin users (a) and non-users (b), respectively, based on the bottom (red), middle (blue), and top (green) VFI tertiles. Survival analysis showed the bottom VFI tertile was associated with significantly better outcomes in metformin non-users but not metformin users.

**

**

**Supplementary Figure S2.** *Univariate Cox proportional models based on metformin use for recurrence free and overall survival in patients belonging to different VFI tertiles.* Univariate Cox proportional model survival curves were generated for 168, 161, and 163 patients belonging to the top (a), middle (b), and bottom (c) VFI tertiles, respectively, based on metformin use (red) and non-use (blue). Survival analysis showed that metformin use was associated with a significantly better recurrence-free survival in patients belonging only to the top tertile.

**Supplementary Table S4**. STROBE Statement–Checklist of items that should be included in reports of cohort studies

| *Item* | | | | *Page^a^* |
| --- | --- | --- | --- | --- |
| *Title and abstract* | 1 | (*a*) Indicate the study’s design with a commonly used term in the title or the abstract | | 1 |
|  |  | (*b*) Provide in the abstract an informative and balanced summary of what was done and what was found | | 3 |
| *Introduction* | | | | |
| Background/rationale | 2 | Explain the scientific background and rationale for the investigation being reported | | 4-5 |
| Objectives | 3 | State specific objectives, including any prespecified hypotheses | | 5 |
| *Methods* | | | | |
| Study design | 4 | Present key elements of study design early in the paper | | 5 |
| Setting | 5 | Describe the setting, locations, and relevant dates, including periods of recruitment, exposure, follow-up, and data collection | | 5 |
| Participants | 6 | (*a*) Give the eligibility criteria, and the sources and methods of selection of participants. Describe methods of follow-up | | 5 |
|  |  | (*b*) For matched studies, give matching criteria and number of exposed and unexposed | | NA |
| Variables | 7 | Clearly define all outcomes, exposures, predictors, potential confounders, and effect modifiers. Give diagnostic criteria, if applicable | | 5-7 |
| Data sources/ measurement | 8* | For each variable of interest, give sources of data and details of methods of assessment (measurement). Describe comparability of assessment methods if there is more than one group | | 5-7 |
| Bias | 9 | Describe any efforts to address potential sources of bias | | 7 |
| Study size | 10 | Explain how the study size was arrived at | | NA |
| Quantitative variables | 11 | Explain how quantitative variables were handled in the analyses. If applicable, describe which groupings were chosen and why | | 6-7 |
| Statistical methods | 12 | (*a*) Describe all statistical methods, including those used to control for confounding | | 7 |
|  |  | (*b*) Describe any methods used to examine subgroups and interactions | | 7 |
|  |  | (*c*) Explain how missing data were addressed | | NA |
|  |  | (*d*) If applicable, explain how loss to follow-up was addressed | | NA |
|  |  | (*e*) Describe any sensitivity analyses | | NA |
| *Results* | | | |  |
| Participants | 13* | (a) Report numbers of individuals at each stage of study—eg numbers potentially eligible, examined for eligibility, confirmed eligible, included in the study, completing follow-up, and analysed | | 7, 10- 11 |
|  |  | (b) Give reasons for non-participation at each stage | | NA |
|  |  | (c) Consider use of a flow diagram | | NA |
| Descriptive data | 14* | (a) Give characteristics of study participants (eg demographic, clinical, social) and information on exposures and potential confounders | | 7-8 |
|  |  | (b) Indicate number of participants with missing data for each variable of interest | | 17 |
|  |  | (c) Summarise follow-up time (eg, average and total amount) | | 8 |
| Outcome data | 15* | Report numbers of outcome events or summary measures over time | | 8 |
| Main results | 16 | (a) Give unadjusted estimates and, if applicable, confounder-adjusted estimates and their precision (eg, 95% confidence interval). Make clear which confounders were adjusted for and why they were included | | 8-10 |
|  |  | (b) Report category boundaries when continuous variables were categorized | | 7 |
|  |  | (c) If relevant, consider translating estimates of relative risk into absolute risk for a meaningful time period | | NA |
| Other analyses | 17 | Report other analyses done—eg analyses of subgroups and interactions, and sensitivity analyses | | 10-11 |
| *Discussion* | | | | |
| Key results | 18 | | Summarise key results with reference to study objectives | 11 |
| Limitations | 19 | | Discuss limitations of the study, taking into account sources of potential bias or imprecision. Discuss both direction and magnitude of any potential bias | 12 |
| Interpretation | 20 | | Give a cautious overall interpretation of results considering objectives, limitations, multiplicity of analyses, results from similar studies, and other relevant evidence | 11-12 |
| Generalisability | 21 | | Discuss the generalisability (external validity) of the study results | 12 |
| *Other information* | | | | |
| Funding | 22 | | Give the source of funding and the role of the funders for the present study and, if applicable, for the original study on which the present article is based | 12 |

^a^ Page numbers refer to the submitted manuscript document.

STROBE, Strengthening the Reporting of Observational Studies in Epidemiology.

**Supplementary Text S1.**

The CT image analysis was performed by authors YRV and SD on CT scans obtained from the institutional PACS database. Prior to the commencement of the image analysis of the study dataset, YRV and SD analyzed a set of 54 CT scans already segmented by ABACS+ to check for interobserver reliability. The Pearson R^2^ correlation and Interclass correlation coefficients for the measurements between them were 0.99 (p < 0.001) and 0.99 (p < 0.001) showing excellent interobserver reliability. Additionally, we performed Bland Altman analysis to understand interobserver variation and found an average variation of 0.9% (SD = 3.57%) between the two measurements for the whole dataset.
